# The aetiology, clinical presentation and treatment of patients with pulmonary hypertension in Cape Town: A preliminary report from the Groote Schuur Hospital Pulmonary Hypertension Registry

**DOI:** 10.7196/AJTCCM.2018.v24i4.218

**Published:** 2018-12-20

**Authors:** S Davies-van Es, G Calligaro, K Manning, H Williams, K Dheda, G Symons

**Affiliations:** 1 Department of General Medicine, Groote Schuur Hospital, Cape Town, South Africa; 2 Division of Pulmonology, Department of Medicine, Groote Schuur Hospital, Cape Town, South Africa

**Keywords:** pulmonary hypertension, South Africa, Pulmonary Hypertension Registry, HIV-associated pulmonary hypertension, chronic thromboembolic pulmonary hypertension, pulmonary arterial hypertension

## Abstract

**Background:**

There is a paucity of knowledge about pulmonary hypertension (PH) in sub-Saharan Africa and an urgent need for its investigation in this context. The impact of HIV infection in PH is also unknown.

**Objectives:**

To determine the aetiology, clinical presentation, severity and current management of PH at a tertiary-level hospital in Cape Town, South Africa (SA).

**Methods:**

Demographic and clinical data, including from special investigations, were captured retrospectively for all patients referred to the Groote Schuur Hospital Pulmonary Hypertension Clinic between October 2015 and November 2017 (n=58) and entered into an online registry. Descriptive statistics were used to present the baseline data at enrolment.

**Results:**

Patients were mainly young and female and almost half (48.3%) had severe symptoms according to World Health Organization classification. The main aetiologies were pulmonary arterial hypertension (PAH) and chronic thromboembolic PH. More than a fifth of the patients were HIV-positive, with nine patients presenting with HIV-associated PAH. The median time from initial presentation to referral to a specialist centre was 227 days (interquartile range: 72 - 625 days). Only a small number of patients were on PH-specific treatment at enrolment and a notable number never underwent right-heart catheterisation.

**Conclusion:**

PH diagnosis is often delayed and even at a tertiary institution with a dedicated clinic and access to special investigations, PH is suboptimally investigated and managed. Expansion of this registry to better understand the phenotype of this disease in SA can improve outcomes for these patients through awareness, early identification and effective management.

## Background

Pulmonary hypertension (PH) is a debilitating and progressive life-threatening disease. PH is characterised by an increase of ≥25 mmHg in mean pulmonary artery pressure (mPAP) at rest (as measured during right-heart catheterisation (RHC)),^[1]^ leading to effort intolerance, right-heart failure and ultimately premature death. PH is classified into five groups by the World Health Organization (WHO) based on aetiology:^[2]^

- Group 1: Pulmonary arterial hypertension (PAH)
- Group 2: Pulmonary hypertension due to left-heart disease (PHLHD)
- Group 3: Pulmonary hypertension due to lung diseases and/or hypoxia (PH-LD)
- Group 4: Chronic thromboembolic pulmonary hypertension (CTEPH)
- Group 5: PH with unclear and/or multifactorial mechanisms.

There is a paucity of knowledge on the prevalence, severity, aetiology, presentation and outcome of PH in sub-Saharan Africa. The PanAfrican Pulmonary Hypertension Cohort (PAPUCO) registry has provided some insights, highlighting the different aetiologies of PH in Africa (including interactions with infectious diseases such as HIV infection, tuberculosis and schistosomiasis and the contribution from uncorrected congenital heart disease) and exposing the poor access to investigative strategies for this condition and the lack of research on affordable treatment.^[3]^ Most other African studies have focused on specific subgroups of PH, such as PH related to sickle cell anaemia^[4]^ and PH related to schistosomiasis.^[5]^ There is an urgent need to study PH in the South African (SA) context, especially given the high prevalence of HIV in this setting,^[6]^ which is known to be a risk factor for the development of PAH.^[7]^

The Pulmonary Hypertension Clinic and Registry at Groote Schuur Hospital (GSH), Cape Town, were set up in 2015, in partnership with the Jenna Lowe Trust. Jenna Lowe was a young woman affected by PH and was a vocal and outspoken advocate for the awareness and treatment of PH in SA. Following her death, the trustees have continued to work to raise the profile of and support research in both PH and solid-organ transplantation. Similar international registries, such as those in the USA,^[8]^ France^[9]^ and Switzerland^[10]^ have been instrumental in characterising patients with this disease, describing its natural history and providing insight into outcomes of patients on PH-specific treatment. This preliminary report serves to highlight some of the findings from the Groote Schuur Hospital Pulmonary Hypertension Registry (GSHPHR), with the aim of better understanding the aetiology, clinical presentation, severity and current management of PH in SA.

## Methods

All patients referred to the GSH Pulmonary Hypertension Clinic (PHC) from October 2015 to November 2017 were eligible for inclusion into the registry. Demographic and clinical data, as well as data from special investigations, were captured retrospectively from medical records. Clinical data included: PH group; presenting symptoms; pre-existing medical conditions; current treatment; risk factors for PH; current WHO functional class; date of onset of initial symptoms, and whether or not an initial diagnosis other than PH was considered. Relevant findings from the clinical examinations were recorded. Special investigations captured included results of: chest radiographs and other imaging; electrocardiograms; pulmonary function tests; 6-minute walk tests; echocardiograms; RHCs, and blood investigations.

Ethical approval was granted by the University of Cape Town’s Human Research Ethics Committee (ref. no. R008/2016 and ref. no. 205/2018); necessary approval was also obtained from treating facilities. Data were captured via an online registry using REDCap, a web-based data collection tool that is compliant with good clinical practice. The registry is password-protected and was accessible only to the specified investigators.

Descriptive statistics were used to present the baseline data from enrolment into the registry. Continuous variables were summarised as medians with their associated interquartile ranges (IQRs) and categorical variables were presented as frequencies and percentages.

## Results

### Demographics

Data were captured from 58 patients under the care of the PHC. The majority were female (n=46; 79%) and the mean (SD) age at enrolment was 44 (16) years. 

### Aetiology of pulmonary hypertension

**Fig. 1:**
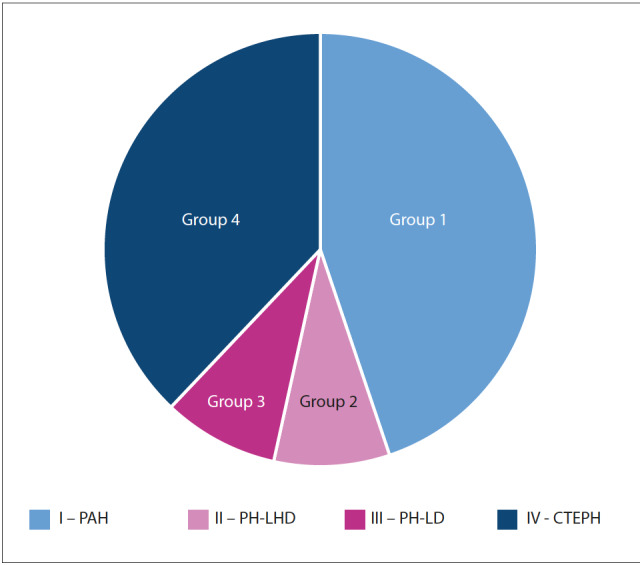
Aetiology of pulmonary hypertension according to classification by the World Health Organization. PAH = pulmonary arterial hypertension PH-LHD = pulmonary hypertension due to left-heart disease PH-LD = pulmonary hypertension due to lung disease and/or hypoxia CTEPH = chronic thromboembolic pulmonary hypertension

Of the total number of patients, 26 (44.8%) were classified as Group1, five (8.6%) as Group 2, five (8.6%) as Group 3, and 22 (37.9%) as Group 4 (Fig. 1). There were no patients classified as Group 5. All patients classified as Group 1 had pulmonary thromboemboli excluded by either a ventilation–perfusion scan (n=20; 76.9%) or computed tomography pulmonary angiography (n=6; 23.1%), and almost all had RHC to confirm the diagnosis (n=23; 88.5%).

**Fig. 2:**
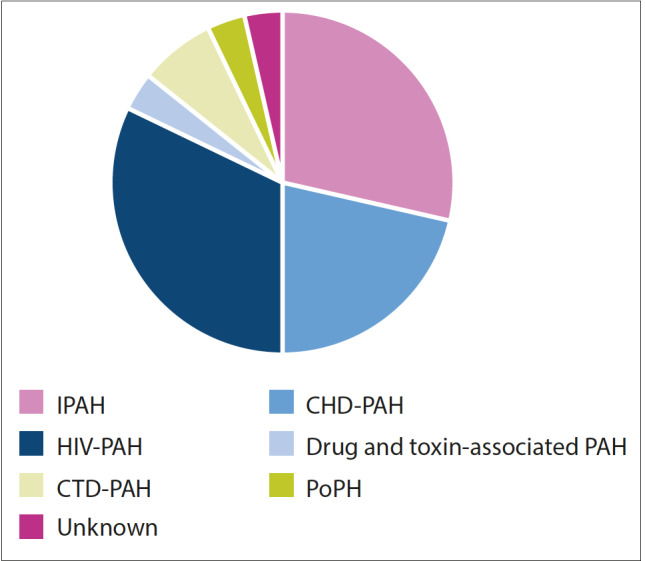
Subgroups of pulmonary arterial hypertension. PAH = pulmonary arterial hypertension IPAH = idiopathic PAH HIV-PAH = PAH associated with HIV infection CHD-PAH = PAH associated with chronic heart disease CTD-PAH = PAH associated with connective tissue disease PoPH = portopulmonary hypertension

Of the patients in Group 1, eight (30.8%) had idiopathic pulmonary arterial hypertension (IPAH), six (23.1%) had PAH associated with connective tissue disease, nine (34.6%) had HIV-associated PAH, one (3.8%) had portopulmonary hypertension, two (7.7%) had PAH related to congenital heart disease and one (3.8%) had drug- and toxin-associated PAH (Fig. 2). Subgroup classification was unknown for one of these patients (3.8%). 

### Presenting symptoms

**Fig. 3:**
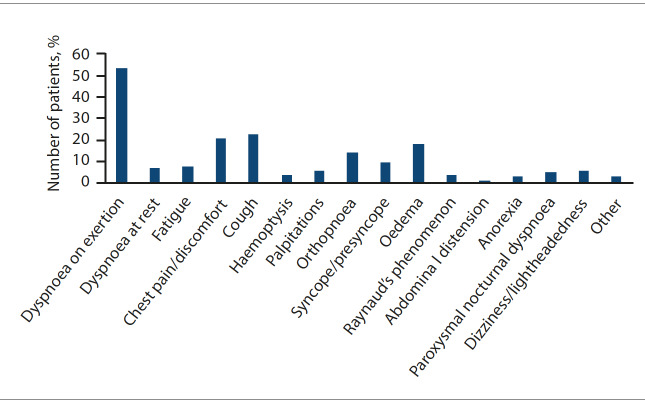
Initial symptoms experienced by pulmonary hypertension patients.

Presenting symptoms were recorded for 57 patients. Almost all of these patients had dyspnoea on exertion as one of their initial presenting symptoms (n=53; 93.0%). Other common presenting symptoms included chest pain (n=21; 36.8%), cough (n=23; 40.4%) and oedema (n=18; 31.6%) (Fig. 3).

### Functional status

**Fig. 4:**
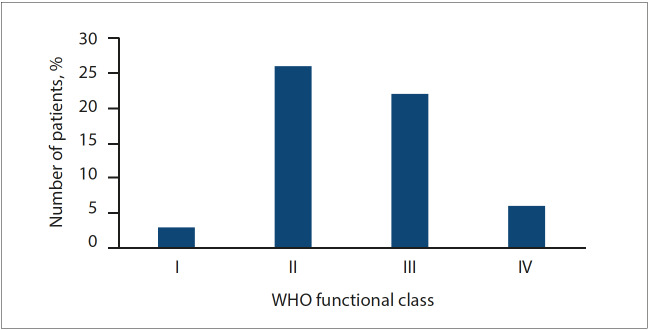
Functional status of patients at presentation according to World Health Organization classification.

The WHO functional class at enrolment was recorded for 57 patients. It was noted that three patients (5.3%) were described as class I, 26 (45.6%) as class II, 22 (38.6%) as class III and six (10.5%) as class IV (Fig. 4). In the PAH group (Group 1), 17 patients (65.4%) were described as functional class III or IV.

### Initial diagnoses other than pulmonary hypertension and time to clinic referral

**Fig. 5:**
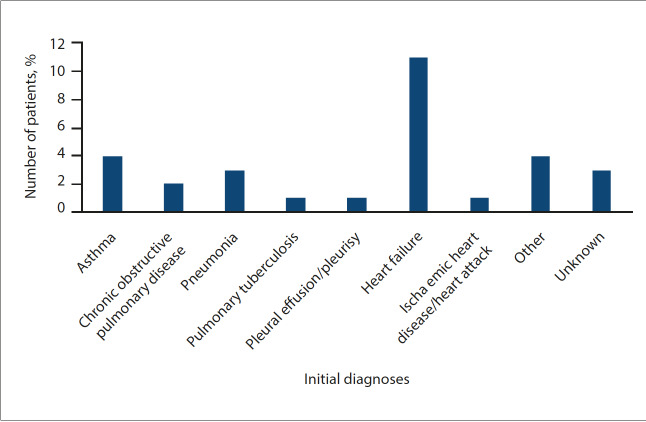
Initial diagnoses, other than pulmonary hypertension, at presentation.

When patients initially presented with symptoms to a healthcare provider, a diagnosis other than PH was made in 30 cases (51.7%). In eight cases (13.8%) it was unknown whether another initial diagnosis was made. The most common of these initial diagnoses were heart failure (n=11; 36.7%), asthma (n=4; 13.3%) and pneumonia (n=3; 10.0%) (Fig. 5). From the records of 48 patients, the median (IQR) time from initial presentation to a healthcare provider to enrolment at the PHC was 227 (72 – 625) days.

### Coexisting medical conditions

The most common coexisting medical conditions were hypertension (n=22; 37.9%), pulmonary embolus (n=18; 31%) and HIV infection (n=13; 22.4%). The full list can be found in Appendix 1 (http://www.ajtccm.org.za/public/docs/GSHPHR_DATATABLE_ADDENDUM.pdf).

**HIV infection**

More than one-fifth (n=13; 22.4%) of the cohort is HIV-positive, all of whom were on antiretroviral treatment at the time of enrolment. In seven cases (53.8%) the viral load was noted to be suppressed, whereas it was either unsuppressed or not documented in the six remaining cases. The median (IQR) CD4 count was 560 (260 - 709) cells/µL. The majority of these HIV-positive patients (n=9; 69.2%) had HIV-associated PAH. The remaining cases included two patients (15.4%) with CTEPH, one (7.7%) with portopulmonary hypertension and one (7.7%) with PAH associated with congenital heart disease (atrial septal defect).

**Chronic thromboembolic pulmonary hypertension**

Of the 22 patients with CTEPH, three (13.6%) had previously received thrombolysis, three (13.6%) had an inferior vena cava filter and five (22.7%) had had a pulmonary thromboendarterectomy.

### Specific treatment for pulmonary hypertension

Only five patients were on PH-specific treatment at the time of enrolment into the clinic, with three classified as Group 1 (11.5%) and two as Group 4 (9.1%). Four of the patients were on monotherapy (sildenafil only), whereas one patient (with HIV-PAH) was on dual therapy (sildenafil and bosentan). 

### Risk factors

None of the patients were documented to have a positive family history of PH. One patient (2% of 51 captured cases) was documented to have a history of anorexigen use, although this was not the aetiology of PH. Two patients (3.9% of 52 captured cases) had a history of substance abuse.

**Smoking**

A positive smoking history was recorded for 20 patients (34.5%), with a median (IQR) of 15 (4 - 30) pack years.

### Echocardiography

History of an echocardiogram prior to being referred to the clinic was recorded in 49 out of 56 patients (87.5%). Owing to difficulties in standardising the reporting of pulmonary artery pressure during echocardiography, comparable reports of the systolic pulmonary artery pressure were available in only 12 of these records, with a median (IQR) reading of 64.5 (61.0 - 77.5) mmHg. The ejection fraction was recorded in 27 of the patients, with a median (IQR) of 64% (55 - 71%). Annotated comments in the echocardiogram report specific to PH were noted at the following frequencies: dilated right ventricle in 31 cases (63.3%); dilated right atrium in 28 cases (57.1%); paradoxical wall motion in 6 cases (12.2%), and D-shaped septum in 8 cases (16.3%).

### Right-heart catheterisation

Less than half of the cohort (n=27; 46.6%) had a RHC performed prior to enrolment or during their initial work-up at the clinic; however, this was performed in almost all (88.5%) of the Group 1 patients. Only three of the Group 4 patients (13.6%) and one of the Group 2 patients (20%) and none of the Group 3 patients had had a RHC. Of the three Group 1 patients who did not undergo RHC, one was deemed too sick for the procedure. The reason for the other two patients not undergoing RHC could not be determined. 

The median (IQR) mPAP was 57 (45 - 68) mmHg and the median (IQR) pulmonary vascular resistance was 12.5 (8.4 - 16.6) Wood units. Pulmonary capillary wedge pressure was recorded for 24 of the patients, with a median (IQR) value of 12.5 (9.5 - 18.5) mmHg. For the patients without significant right-to-left shunts in whom cardiac output could be reliably determined, the mean (SD) cardiac output was recorded as 3.48 (1.13) L.

Vasoreactivity testing was performed for 14 of the 27 patients (51.9%): 12 patients were from Group 1 (52.2%), one was from Group 4 (33.3%) and one was from Group 2 (100%). Of these, only one patient (5.9%) with HIV-PAH had a notable fall in mPAP (defined as a decrease of ≥10 mmHg to reach a mean of ≤40 mmHg)^[1]^ following the administration of nitrates. A second patient (with IPAH) had a fall of ≥10 mmHg, but only reached a mean of 63 mmHg. Vasoreactivity testing was performed using intravenous nitrates for all but one of these patients, who received inhaled nitrates during RHC at a private facility. 

### Chest X-ray findings

Chest X-ray findings were recorded for 37 patients. No abnormalities were reported in two (5.4%) of these patients. The most common abnormal findings were enlarged pulmonary artery tracts (n=21; 56.8%), followed by cardiac enlargement (n=12; 32.4%). The full list can be found in Appendix 1.

### Electrocardiogram findings

The analyses of 33 electrocardiograms were captured. The majority (n=26; 78.8%) were in sinus rhythm. Sinus tachycardia was seen in six cases (18.2%), whereas atrial fibrillation was recorded in one case (3%). The most frequently noted abnormal morphology was right ventricular hypertrophy (n=20; 60.6%), followed by right atrial hypertrophy (n=18; 54.5%) and nonspecific ST–T wave changes (n=8; 24.2%), respectively. The full list can be found in Appendix 1.

### Pulmonary function tests

Forced expiratory volume in the first second (FEV1) was recorded in 47 patients, with a median (IQR) of 2.28 (1.74 - 2.78) L. Forced vital capacity (FVC) was recorded in 46 patients, with a median (IQR) of 2.9 (2.42 - 3.36) L. The median FEV1/FVC ratio for these patients was 78.9% (IQR: 73 - 84.7%). A transfer factor for carbon monoxide was recorded for 37 patients. The median (IQR) result was 17.3 (11.73 - 19.67) mL/min/kPa.

### Six-minute walk tests

**Fig. 6:**
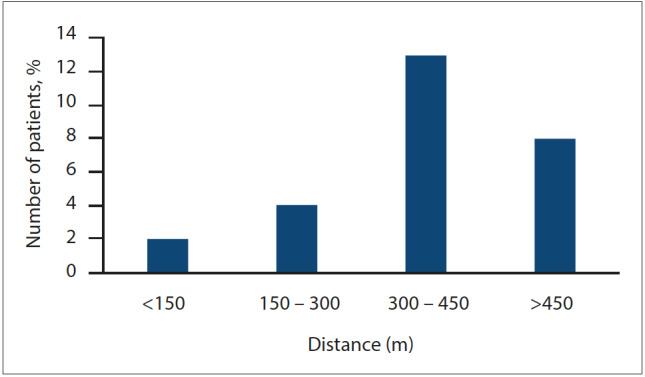
Distance attained during a 6-minute walk test.

A 6-minute walk test was performed in 27 patients (46.6%) at enrolment, 26 of whom had oxygen saturations recorded before and after the test. The median (IQR) distance attained was 400 (330 - 495) m. The median oxygen saturation at rest was 94% (IQR: 91 - 96%) and 93% (IQR: 89 - 97%) after the test. Two of the patients (7.4%) reached <150 m, four (14.8%) managed 150 - 300 m, 13 (48.2%) managed 300 - 450 m and eight (29.6%) managed to walk >450 m (Fig. 6).

## Discussion

PH is an underdiagnosed and under-resourced disease and, to our knowledge, this is the first description of a cohort of SA patients with PH. Like other PH cohorts, our patients were young, predominantly female and almost half had severe symptoms (WHO class III-IV). Very few were on PH-specific treatment at the time of enrolment. In the REVEAL registry,^[11]^ the time from onset of symptoms to diagnosis of PAH was >2 years in 20% of patients, and similar delays in referral and diagnosis were identified in the GSHPHR. This is likely due to the insidious nature and often nonspecific symptoms of this disease. Inherent problems in the SA public health sector, such as a lack of access to diagnostic tests, long waiting times for specialist appointments and a high burden of other infectious and non-infectious diseases,^[12]^ may also complicate the diagnosis of PH.

While the majority of PH patients reported on elsewhere (including in the PAPUCO registry)^[3]^ are classified as Group 2, the patients in the GSHPHR are mainly classified as Group 1 or Group 4. The relative frequency of aetiology of PH reflects referral bias to the PHC at our hospital, with Group 2 or Group 3 patients likely managed at other outpatient clinics.

Previous studies from Switzerland^[13,14]^ and France^[15]^ have shown the prevalence of HIV-associated PAH among HIV-positive patients to be approximately 0.5%. When the burden of HIV in SA is considered, the proportion of patients with HIV-related PH in our cohort is a considerable under-representation. It is unknown whether this is because the burden of HIV-associated PAH is dwarfed by other infectious and non-infectious lung disease, or whether the impact of HIV infection as a single aetiological contributor to the development of PH has been overestimated. In the PAPUCO registry, HIV was found to be a comorbidity more often than it was found to be a causative factor of PH.^[3]^

Owing to limited access to investigations, the work-up of patients in the GSHPHR did not meet standards mandated by international guidelines. While the majority of patients had undergone echocardiography prior to referral, a considerable number never had a RHC performed, either prior to referral or during their initial work-up at the clinic. The European Society of Cardiology/European Respiratory Society (ESC/ERS) guidelines state specifically that RHC is required for the diagnosis of PAH and CTEPH and that echocardiography alone should never be used to initiate specific treatment of PH.^[1]^ Although almost all the Group 1 (PAH) patients underwent RHC, this was not true of patients in the other WHO groups. In the state sector, it is difficult to motivate for cardiac catheterisation if no PH-specific treatment is being proposed, and this is reflected in the small number of patients from groups 2, 3 and 4 who underwent cardiac catheterisation. An additional methodological concern with RHC was that pulmonary capillary wedge pressure was not universally measured, and thus the diagnosis of Group 2 PH was not always conclusively excluded.

ESC/ERS guidelines also state that vasoreactivity testing to assess the benefit of high-dose calcium channel blockers should be performed only for patients with IPAH, hereditary PAH and druginduced PAH.^[1]^ In this cohort, vasoreactivity testing during RHC was not performed consistently for patients according to guideline recommendations, and sometimes it was performed in patients for whom it was not indicated. In addition, vasoreactivity testing was performed almost exclusively with intravenous nitrates, instead of with the recommended inhaled nitric oxide, epoprostenol or intravenous adenosine,^[16]^ as use of these agents in this setting (and even sometimes in the private sector) is prohibitively expensive.

As with many retrospective studies involving review of medical records, the quality of the available information during data capture has been a notable limitation. This problem could be mitigated in future by introducing a standard form for prospective data collection during both enrolment and follow-up visits, including necessary demographic and clinical data, as well as listing the standard investigations expected and how they should be recorded.

As already stated, this was not a cross-sectional study and the relative frequencies of PH aetiologies reflect referral bias to our centre. Currently the registry also consists almost entirely of patients from hospitals in the state sector. Barriers to care among these patients have already been outlined. The reported results may not be generalisable to other state hospitals or the private sector. However, we believe that the inherent limitations to optimal PH care experienced at our institution, which has tried to actively encourage referrals, cohort patients in a specialised PHC, and motivate for pulmonary vasodilators, are likely also prevalent elsewhere in the state and private sectors. As the registry continues to expand and include both private and state patients, the information yielded may become more generalisable.

## Conclusion

Despite deficiencies, this registry provides initial insights into PH and its management in the state sector in SA, particularly regarding PAH and CTEPH. We found that PH diagnosis is often delayed and even at a tertiary institution with a dedicated clinic and access to special investigations, PH is suboptimally investigated and managed compared with international guidelines.

Ultimately, this registry needs to expand to include good-quality data from patients from multiple tertiary-level hospitals in both the private and the public sector in SA. Data collected through such a national registry will help to further the understanding of the phenotype of this disease in SA and through earlier identification, motivation for effective treatment, research and advocacy, hopefully improve outcomes for patients with PH.

## Supplementary Tables

Appendix 1 GSHPHR Data Table

## References

[1] Galiè N, Humbert M, Vachiery J-L (2016). 2015 ESC/ERS Guidelines for the diagnosis and treatment of pulmonary hypertension.. Eur Heart J.

[2] Simonneau G, Gatzoulis MA, Adatia I (2013). Updated clinical classification of pulmonary hypertension.. J Am Coll Cardiol.

[3] Thienemann F, Dzudie A, Mocumbi AO (2016). The causes, treatment, and outcome of pulmonary hypertension in Africa: Insights from the Pan African Pulmonary Hypertension Cohort (PAPUCO) Registry.. Int J Cardiol.

[4] Amadi VN, Balogun MO, Akinola NO, Adebayo RA, Akintomide AO (2017). Pulmonary hypertension in Nigerian adults with sickle cell anemia.. Vasc Heal Risk Manag.

[5] Farrag A, El-Aroussy W, Zaghloul S, El-Guindy M, Yacoub M (2012). Prevalence and severity of pulmonary hypertension in asymptomatic rural residents with schistosomal infection in the Nile Delta.. Trop Med Int Heal.

[6] Statistics South Africa (2017). Mid-year population estimates South Africa, 2017. http://www.statssa.gov.za/publications/P0302/P03022017.pdf.

[7] Opravil M, Pechère M, Speich R (1997). HIV-associated primary pulmonary hypertension. A case control study. Swiss HIV Cohort Study.. Am J Respir Crit Care Med.

[8] McGoon MD, Miller DP (2012). REVEAL: A contemporary US pulmonary arterial hypertension registry.. Eur Respir Rev.

[9] Humbert M, Sitbon O, Chaouat A (2006). Pulmonary arterial hypertension in France: Results from a national registry.. Am J Respir Crit Care Med.

[10] Fischler M, Speich R, Dorschner L (2008). Pulmonary hypertension in Switzerland: Treatment and clinical course.. Swiss Med Wkly.

[11] Brown LM, Chen H, Halpern S (2011). Delay in recognition of pulmonary arterial hypertension: Factors identified from the REVEAL registry.. Chest.

[12] Mayosi BM, Flisher AJ, Lalloo UG, Sitas F, Tollman SM, Bradshaw D (2009). The burden of non-communicable diseases in South Africa.. Lancet.

[13] Speich R, Jenni R, Opravil M, Pfab M, Russi EW (1991). Primary pulmonary hypertension in HIV infection.. Chest.

[14] Zuber J-P, Calmy A, Evison JM (2004). Pulmonary arterial hypertension related to HIV infection: Improved hemodynamics and survival associated with antiretroviral therapy.. Clin Infect Dis.

[15] Sitbon O, Lascoux-Combe C, Delfraissy JF (2008). Prevalence of HIV-related pulmonary arterial hypertension in the current antiretroviral therapy era.. Am J Respir Crit Care Med.

[16] Badesch DB, Abman SH, Simonneau G, Rubin LJ, McLaughlin VV (2007). Medical therapy for pulmonary arterial hypertension: Updated ACCP evidence-based clinical practice guidelines.. Chest.

