# Supplementary material for: The aetiology, clinical presentation and treatment of patients with pulmonary hypertension in Cape Town: A preliminary report from the Groote Schuur Hospital Pulmonary Hypertension Registry
Source: Afr J Thorac Crit Care Med. 2018 Dec 20;24(4):10.7196/AJTCCM.2018.v24i4.218. doi: 10.7196/AJTCCM.2018.v24i4.218 (PMC8424658; doi:10.7196/AJTCCM.2018.v24i4.218)
Supplement: Appendix 1 — GSHPHR Data Table [file AJTCCM-24-4-218-S001.pdf]

Variables	Frequency, *n* (%)
**Demographics (*N*=58)**	
Male	12 (21)
Female	46 (79)
Age (years), mean (SD)	44 (16)
**PH aetiology (*N*=58)**	
- **I – PAH**	26 (44.8)
- IPAH	8 (30.8)
- CTD-associated PAH	6 (23.1)
- HIV-associated PAH	9 (34.6)
- Drug- and toxin-associated PAH	1 (3.8)
- Portopulmonary PAH	1 (3.8)
- PAH related to congenital heart disease	2 (7.7)
- Subgroup unknown	1 (3.8)
- **II – PH secondary to left-heart disease**	5 (8.6)
- Systolic heart failure	1 (20)
- Diastolic heart failure	4 (80)
- **III – PH secondary to pulmonary pathology**	5 (8.6)
- Interstitial lung disease	3 (60)
- Sleep-disordered breathing	1 (20)
- Chronic obstructive pulmonary disease	1 (20)
- **IV – CTEPH**	22 (37.9)
- **V – Miscellaneous**	0

**Initial symptoms (*N*=57)***	**Frequency, *n* (%)**
Dyspnoea on exertion	53 (93)
Dyspnoea at rest	7 (12.3)
Fatigue	8 (14)
Chest pain/discomfort	21 (36.8)
Cough	23 (40.4)
Haemoptysis	4 (7)
Palpitations	6 (10.5)
Orthopnoea	14 (24.6)
Syncope/presyncope	10 (17.5)
Oedema	18 (31.6)
Raynaud's phenomenon	4 (7)
Abdominal distension	1 (1.8)
Anorexia	3 (5.3)
Paroxysmal nocturnal dyspnoea	5 (8.8)
Dizziness/lightheadedness	6 (10.5)
Other	3 (5.3)

**WHO class at presentation (all patients) (*N*=57)***	**Frequency, *n* (%)**
I	3 (5.3)
II	26 (45.6)
III	22 (38.6)
IV	6 (10.5)

**WHO class at presentation (Group 1 – PAH) (*N*=26)**	**Frequency, *n* (%)**
I	1 (3.8)
II	8 (30.8)
III	14 (53.8)
IV	3 (11.5)

**Initial diagnosis other than PH (*N*=58)**	**Frequency, *n* (%)**
Yes	30 (51.7)
- Asthma	- 4 (13.3)
- COPD	- 2 (6.7)
- Pneumonia	- 3 (10)
- PTB	- 1 (3.3)
- Pleural effusion/pleurisy	- 1 (3.3)
- Heart failure	- 11 (36.7)
- Ischaemic heart disease/heart attack	- 1 (3.3)
- Other	- 4 (13.3)
- Unknown	- 3 (10)
No	20 (34.5)
Unknown	8 (13.8)

**Time from initial contact with medical service to enrolment at PHC (days), median (IQR) (*N*=48)***	227 (72 - 625)

**Coexisting medical problems** (*N*=58)	**Frequency, *n* (%)**
Hypertension	22 (37.9)
Diabetes mellitus	5 (8.6)
Ischaemic heart disease	4 (6.9)
Cerebrovascular disease	2 (3.5)
Left ventricular failure	2 (3.5)
HIV	13 (22.4)
Pulmonary embolus	18 (31)
Deep vein thrombosis	5 (8.6)
Solid organ cancer	1 (1.7)
Pulmonary tuberculosis	3 (5.2)
Lupus/SLE	6 (10.3)
Systemic sclerosis/scleroderma	3 (5.2)
Hypothyroidism	2 (3.5)
Chronic kidney disease	1 (1.7)
Chronic obstructive pulmonary disease	1 (1.7)
Asthma	4 (6.9)

Interstitial lung disease	3 (5.2)
Cirrhosis	1 (1.7)
Major depressive disorder	1 (1.7)
Generalised anxiety disorder	1 (1.7)

**HIV-positive patients**, *n* (%)	13 (22.4)
Aetiology of PH in those with HIV (*N*=13)	**Frequency, *n* (%)**
- HIV-PAH	9 (69.2)
- CTEPH	2 (15.4)
- PoPH	1 (7.7)
- CHD-PAH	1 (7.7)
No. on ART	13 (100)
No. virally suppressed on ART	7 (53.8)
No. not virally suppressed or with unknown viral load	6 (46.2)
CD4 (cells/µL), median (IQR)	560 (260 – 709)

**Patients with CTEPH (*N*=22)**	**Frequency, *n* (%)**
- Received thrombolysis	3 (13.6)
- Have an IVC filter	3 (13.6)
- Have had a thrombectomy	5 (22.7)

**Number of patients on PH specific treatment at enrolment**	**Frequency, *n* (%)**
Group 1 – PAH (*N*=26)	3 (11.5)
- Sildenafil	2 (7.7)
- Sildenafil + bosentan	1 (3.8)
Group 4 – CTEPH (*N*=22)	2 (9.1)
- Sildenafil	2 (9.1)

**Risk factors**	**Frequency, *n* (%)**
Positive family history of PH (*N*=58)	0
Positive history of anorexigen use (*N*=51)*	1 (2)
Positive history of substance use (*N*=52)*	2 (3.9)

**Smoking history** (*N*=58)	**Frequency, *n* (%)**
Yes	20 (34.5)
- Median pack years (IQR)	15 (4 - 30) PY
No	27 (46.6)
Unknown	11 (19)

**Echocardiography**	**Frequency, *n* (%)**
No. of patients who had echo prior to referral (*N*=56)*	49 (87.5)
Dilated RV	31 (63.3)
Dilated RA	28 (57.1)
Paradoxical wall motion	6 (12.2)
D-shaped septum	8 (16.3)

Systolic PAP (mmHg), median (IQR) (*N*=12)*	64.5 (61 - 77.5)
EF% median (IQR) (*N*=27)*	64% (55 - 71%)

**Diagnostic right heart catheterization (RHC)**	**Frequency, *n* (%)**
No. of patients who had RHC (*N*=58)	27 (46.6)
- Group 1 (PAH) (*N*=26)	23 (88.5)
- IPAH (*N*=8)	7 (87.5)
- HIV-PAH (*N*=9)	8 (88.9)
- CTD-PAH (*N*=6)	4 (66.7)
- CHD-PAH (*N*=2)	2 (100)
- Drug and toxin PAH (*N*=1)	1 (100)
- Group 2 (PH-LHD) (*N*=5)	1 (20)
- Group 3 (PH-LD) (*N*=5)	0
- Group 4 (CTEPH) (*N*=22)	3 (13.6)
Mean PAP (mmHg), median (IQR) (*N*=27)	57 (45 – 68)
Pulmonary vascular resistance (Wood units), median (IQR) (*N*=23)*	12.5 (8.4 - 16.6)
Cardiac output (L), mean (SD) (*N*=21)*	3.48 (1.13)
Pulmonary capillary wedge pressure (mmHg), median (IQR) (*N*=24)*	12.5 (9.5 - 18.5)
Vasoreactivity testing with nitrates performed (*N*=27)	14 (51.9)
- Group 1 (PAH) (*N*=23)	12 (52.2)
- IPAH (*N*=7)	3 (42.9)
- HIV-PAH (*N*=8)	5 (62.5)
- CTD-PAH (*N*=4)	3 (75)
- CHD-PAH (*N*=2)	0
- Drug and toxin PAH (*N*=1)	1 (100)
- Group 2 (PH-LHD) (*N*=1)	1 (100)
- Group 4 (CTEPH) (*N*=3)	1 (33.3)
Number who had a fall of at least 10 mmHg to an absolute value of ≤40 mmHg after nitrates (*N*=17)	1 (5.9)

**Chest X-ray findings** (*N*=37)*	**Frequency, *n* (%)**
No abnormalities	2 (5.4)
Interstitial lung disease	1 (2.7)
Enlarged pulmonary artery tracts	21 (56.8)
Hyperinflation/COPD	1 (2.7)
Cardiac enlargement	12 (32.4)

**ECG findings** (*N*=33)*	**Frequency, *n* (%)**
Rhythm	
- Normal sinus rhythm	26 (78.8)
- Sinus tachycardia	6 (18.2)
- Atrial fibrillation	1 (3)

Morphology	
- No abnormalities	1 (3)
- Right ventricular hypertrophy	20 (60.6)
- Left ventricular hypertrophy	1 (3)
- Right atrial hypertrophy	18 (54.5)
- Right bundle branch block	7 (21.2)
- ST depression	6 (18.2)
- Non-specific ST–T wave changes	8 (24.2)
- Q waves	1 (3)
- Other	7 (21.2)

**Pulmonary function testing**	
FEV1 (L), median (IQR) (*N*=47)*	2.28 (1.74 - 2.78)
FVC (L), median (IQR) (*N*=46)*	2.9 (2.42 - 3.36)
FEV1/FVC ratio, median (IQR) (*N*=46)*	78.9% (73 - 84.7%)
TLCO (mL/min/kPa), median (IQR) (*N*=37)*	17.3 (11.73 - 19.67)

**6-minute walk test (6MWT)**	**Frequency, *n* (%)**
No. of patients who had 6MWT (*N*=58)	27 (46.6)
Distance attained pre-treatment (m), median (IQR) (*N*=27)*	400 (330 - 495)
No. <150 m	2 (7.4)
No. 150 - 300 m	4 (14.8)
No. 300 - 450 m	13 (48.2)
No. >450 m	8 (29.6)
Pulse oximetry at rest, median (IQR) (*N*=26)*	94% (91 - 96%)
Pulse oximetry at end of 6MWT, median (IQR) (*N*=26)*	93% (89 - 97%)

*Fewer than 58 patients (total cohort) owing to missing data.
